# Exploring the role and mechanism of Astragalus membranaceus and radix paeoniae rubra in idiopathic pulmonary fibrosis through network pharmacology and experimental validation

**DOI:** 10.1038/s41598-023-36944-1

**Published:** 2023-09-04

**Authors:** Huanyu Jiang, Rui Zhou, Liping An, Junfeng Guo, Xinhui Hou, Jiao Tang, Fei Wang, Quanyu Du

**Affiliations:** 1 Department of Geriatrics, Hospital of Chengdu University of Traditional Chinese Medicine, Chengdu, 610072 Sichuan China; 2 School of Basic Medical Sciences, Chengdu University of Traditional Chinese Medicine, Chengdu, 611130 Sichuan China; 3 Department of Endocrinology, Hospital of Chengdu University of Traditional Chinese Medicine, Chengdu, 610072 Sichuan China

**Keywords:** Pharmacology, Respiratory tract diseases

## Abstract

Idiopathic pulmonary fibrosis (IPF) is a chronic, progressive fibrotic disease with an unclear etiology and no effective treatment. This study aims to elucidate the pathogenic mechanism networks involving multiple targets and pathways in IPF. Extracts and metabolites of Astragalus membranaceus (AM) and Radix paeoniae rubra (RPR), two well-known traditional Chinese medicines, have demonstrated therapeutic effects on IPF. However, the underlying mechanisms of AM and RPR remain unclear. Utilizing network pharmacology analysis, differentially expressed genes (DEGs) associated with IPF were obtained from the GEO database. Targets of AM and RPR were identified using the TCM Systems Pharmacology Database and Analysis Platform and SwissTargetPrediction. A protein–protein interaction (PPI) network was subsequently constructed and analyzed using the STRING database and Cytoscape software. Gene ontology enrichment analysis and kyoto encyclopedia of genes and genomes analysis were conducted using Metascape. Additionally, a component-target-pathway network and a Sankey diagram were employed to identify the main active components, and molecular docking was performed between these components and proteins encoded by key targets. Finally, in vivo studies were conducted based on network pharmacology. A total of 117 common targets between DEGs of IPF and drug targets were identified and included in the PPI network, in which *AKT1*, *MAPK3*, *HSP90AA1*, *VEGFA*, *CASP3*, *JUN*, *HIF1A*, *CCND1*, *PTGS2*, and *MDM2* were predicted as key targets. These 117 targets were enriched in the PI3K-AKT pathway, HIF-1 signaling pathway, apoptosis, and microRNAs in cancer. Astragaloside III, (R)-Isomucronulatol, Astragaloside I, Paeoniflorin, and β-sitosterol were selected as the main active components. Docking scores ranged from − 4.7 to − 10.7 kcal/mol, indicating a strong binding affinity between the main active compounds and key targets. In vivo studies have indeed shown that AM and RPR can alleviate the pathological lung fibrotic damage caused by bleomycin treatment. The treatment with AM and RPR resulted in a reduction of mRNA levels for key targets *AKT1*, *HSP90AA1*, *CASP3*, *MAPK3*, and *VEGFA*. Additionally, the protein expression levels of *AKT1*, *HSP90AA1*, and *VEGFA* were also reduced. These results support the therapeutic potential of AM and RPR in ameliorating pulmonary fibrosis and provide insight into the molecular mechanisms involved in their therapeutic effects.

## Introduction

Pulmonary fibrosis (PF) results from various interstitial lung diseases, with idiopathic pulmonary fibrosis (IPF) constituting the majority of cases^1^. IPF, a subtype of idiopathic interstitial pneumonia (IIP), is characterized by diffuse alveolitis and alveolar structural disorders, primarily affecting adults over 50 years old^2^. The main clinical manifestations include chronic, progressively worsening dyspnea, lung functions, and functional capacities. IPF is associated with poor survival and prognosis, exhibiting a median survival time of 3–5 years post-diagnosis^3^. Patients may succumb to respiratory failure, acute exacerbation, pulmonary hypertension, lung cancer, or other complications^4^. IPF incidence ranges from 2 to 29 per 100,000, as indicated by different studies^5,6^. Currently, there is no nationwide epidemiological data for IPF in Mainland China. Regional data from several large samples reveal that the incidence of interstitial lung disease (ILD) is significantly increasing in China. Pirfenidone and Nintedanib are the only treatment agents for IPF recommended by ATS/ERS/JRS/ALAT^7^. However, these agents mainly reduce the decline rate of lung functions but cannot reverse progressive deterioration or improve survival rates^8–11^. Adverse events and high medical costs must also be considered in clinical practice.

Traditional Chinese Medicine (TCM) has increasingly been adopted as a complementary and alternative medicine worldwide. Chinese herbal formulas are the most commonly used TCM treatment in China, and have demonstrated positive effects on improving the quality of life and alleviating symptoms of ILD patients^12,13^. Consequently, Chinese herbal medicines, with their thousands of years of history, are excellent candidates for exploring and evaluating IPF treatment. IPF can be classified as “the atrophic lung disease” in TCM due to its clinical characteristics, such as dyspnea, chronic cough, shortness of breath, and expectoration.

Astragalus membranaceus (AM), also known as "Huangqi," is a Chinese herb that has been utilized by TCM practitioners for over two thousand years^14^. Over 100 active metabolites of AM, including flavonoids, saponins, polysaccharides, and amino acids, have been identified^15^. Numerous studies have demonstrated the antiviral, immunomodulatory, and anti-inflammatory properties of these metabolites both in vivo and in vitro^16–18^. Radix paeoniae rubra (RPR), also known as Chishao in China, is the dried root of *Paeonia lactiflora* Pall. or *Paeonia veitchii* Lynch^19^. Active ingredients of RPR, such as flavonoids, monoterpene, tannins, and phenolic acids, exhibit a variety of biological activities, including anti-inflammation, immunoregulation, antiviral, and antiallergic properties^20–22^. Some studies have suggested that certain components of AM and RPR may have therapeutic roles in IPF.

**Figure 1:**
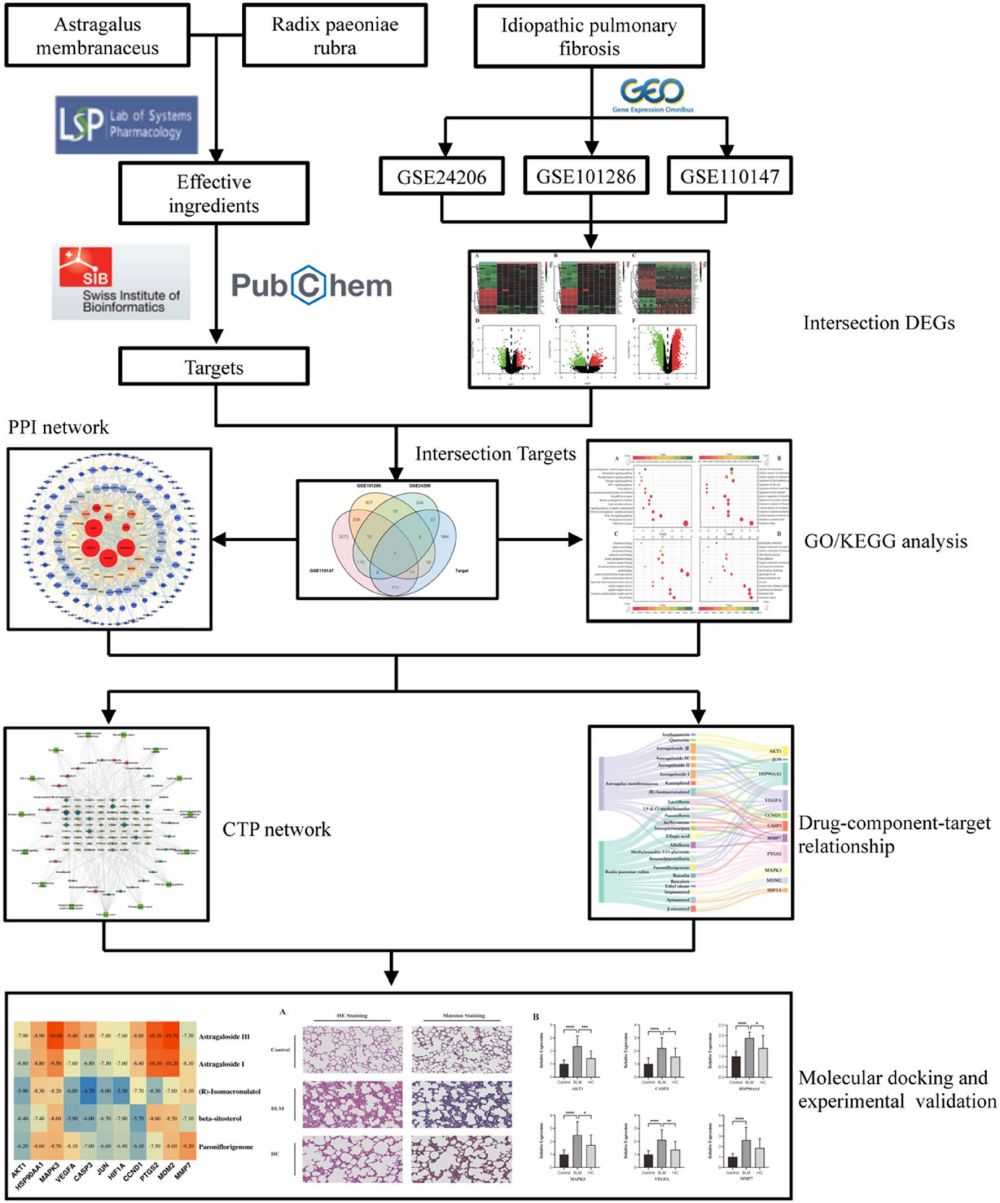
Technical strategy of the study.

The compatibility of Chinese herbs forms the basis of TCM for disease treatment, and the improved pharmacological effects of medicine pairs often result from the synergistic effect of different components with specific pharmacokinetic characteristics. However, the multi-ingredient and multi-target characteristics of herbs make identifying their molecular mechanisms difficult. Consequently, computer-aided identification methods, represented by network pharmacology and molecular docking, were employed in this study to predict the potential mechanisms of AM and RPR on IPF. A bleomycin (BLM)-induced pulmonary fibrosis rat model was then utilized to verify their therapeutic effects. The technical strategy of the study is illustrated in Fig. 1.

## Materials and methods

### Differentially expressed genes in IPF

We downloaded 17 tissues (11 lung tissues with IPF and 6 control lung tissues) from GSE24206 (GPL570 platform), 10 tissues (7 lung tissues with IPF and 3 control lung tissues) from GSE101286 (GPL6947 platform), and 33 tissues (22 lung tissues with IPF and 11 control lung tissues) from GSE110147 (GPL6244 platform) from the GEO database (https://www.ncbi.nlm.nih.gov/geo/, 6/17/2022)^23^. The three data sets were read and standardized using the “Affy” and “Oligo” packages. Differentially expressed genes (DEGs) between IPF and control tissues were identified using the “limma” package, applying a threshold of |logFC|> 0.5 and FDR < 0.05.

### Bioactive ingredients and targets screening

The Traditional Chinese Medicine Database and Analysis Platform (TCMSP, https://tcmspw.com/tcmsp.php, 6/17/2022) was utilized to determine the chemical components of AM and RPR based on two ADME parameters: oral bioavailability (OB) and drug-likeness (DL).^24^ Components that fulfilled the criteria of OB ≥ 30% and DL ≥ 0.18 were considered active components and downloaded from TCMSP for further analysis. Additionally, metabolites that did not meet the aforementioned screening criteria but possessed potential activity in IPF treatment were included by searching and reviewing relevant literature on PubMed, CNKI, and Web of Science. The 2D molecular structure files (.SDF) of active components were downloaded from the PubChem database (https://pubchem.ncbi.nlm.nih.gov/, 6/17/2022) and submitted to SwissTargetPrediction (http://www.swisstargetprediction.ch/) with the species setting as “Homo Sapiens” to predict targets^25,26^. Ultimately, potential targets of each active component with a probability over 0.3 were retained.

The overlapping part of DEGs between IPF and control tissues and the targets of AM and RPR were obtained using a Venn diagram. Moreover, overlapping targets were imported into the STRING database (version 11.5) (https://www.string-db.org/, 6/17/2022) with the species setting as “Homo sapiens” and confidence level > 0.4, to construct a protein–protein interaction (PPI) network that reflected the physical and functional interactions between proteins.^27^ Results in the. tsv format were introduced into Cytoscape v3.7.2 to visualize the data and extract the hub genes according to the degree.

### GO/KEGG enrichment analyses

The Gene Ontology (GO) and Kyoto Encyclopedia of Genes and Genomes (KEGG) enrichment analysis were conducted using Metascape (https://metascape.org/, 6/17/2022)^28^. Bio-enrichment results for these genes, encompassing molecular functions (MF), cellular components (CC), biological processes (BP), and signaling pathways, were obtained through this process. In this study, we selected the top 15 enrichment results based on the *P*-value and utilized bubble charts to visualize the results. The component-target-pathway (CTP) network was constructed based on target-component results acquired from SwissTargetPrediction and target-pathway results derived from KEGG analysis.

### Molecular docking

Proteins encoded by the 10 key genes and the DEG, which were significant in three data sets, were selected as macromolecular receptors. Active components that were predicted to have a close relationship with the 11 genes were chosen as ligands. We then visualized the relationship between these components and genes using a Sankey diagram. The 2D structure files of active components downloaded from PubChem were converted into PDB files using Chem3D software (version 18.0). The 3D crystal structure files of proteins were downloaded from the Worldwide Protein Data Bank (wwPDB, https://www.rcsb.org/, 6/18/2022), and all PDB files were converted to PDBQT files using AutoDock Tool (version 4.2) for further molecular docking in Autodock Vina^29,30^. Finally, the binding affinity, represented by the docking score.

### Model preparation and administrations

This study was approved by the Animal Ethics Committee of Chengdu University of Traditional Chinese Medicine, with the ethical approval number 2022–48. Throughout the experimental process, we strictly adhered to international and national animal welfare guidelines and the ARRIVE guidelines.

Specific pathogen-free SD male rats (weight, 180 ± 20 g) were purchased from Chengdu Dashuo Experimental Animal Co., LTD. (Chengdu, China). After acclimating to experimental conditions for one week, all the animals were randomly divided into three groups: Blank control group (Control), Bleomycin model group (BLM), and Huangqi-Chishao group (HC), with 6 rats in each group. The BLM and HC groups were injected with Bleomycin sulfate (Cat No. S1214, Selleck, Houston, TX, United States) 5 mg/kg by endotracheal intubation to construct the pulmonary fibrosis rat model^32^. The control group was treated with an intratracheal injection of the same volume of normal saline. Gavage treatment began on the second day after bleomycin injection and continued for 28 days. The HC group received 8.2 g/kg of traditional Chinese medicine decoction (AM 30 g and RPR 10 g) purchased from Sichuan New Green Pharmaceutical Technology Development Co., Ltd. (Chengdu, China). The control and BLM groups were given the same volume of normal saline. Rats were sacrificed 8 h after the final administration. Rats were anesthetized with an intraperitoneal injection of sodium pentobarbital (30 mg/kg), followed by laparotomy to expose the abdominal cavity. The rats were then euthanized through exsanguination from the abdominal aorta, and lung tissues were collected via thoracotomy. The left lung was fixed in 4% paraformaldehyde for 24 h and then embedded in paraffin for histological analysis and immunohistochemical detection, while the right lung was cryopreserved for colorimetric assay and qPCR.

### Colorimetric assay

The concentrations of HYP in the lung tissues were measured using a colorimetric assay kit (E-BC-K602-M, Elabscience, Wuhan, China). 100 mg lung tissues were collected, minced, and combined with 1 mL of 6 mol/L hydrochloric acid solution, followed by hydrolysis at 95 °C for 6 h. The pH of the hydrolyzed sample was adjusted to 6.5–7.0. A 2 mL aliquot of the hydrolyzed sample was mixed with 20 mg of Clarificant, centrifuged at 1500 rpm for 10 min, and 400 μL of the supernatant was collected for analysis. To the 400 μL of standard and test samples, 200 μL of Oxidant Agent was added, left to stand for 15 min at room temperature, followed by the addition of 400 μL of Chromogenic Agent. The samples were then incubated in a 60 ℃ water bath for 15 min before being transferred to an enzyme-linked immunosorbent assay plate. The optical density values at 558 nm were measured using an ELISA reader.

### RNA extraction, reverse transcription, and qPCR

Total RNA in lung tissues was extracted using the Animal Total RNA Isolation Kit (RE-03014, Foregene, Chengdu, China). After determining the RNA concentration with a NanoDrop spectrophotometer, reverse transcription was performed to synthesize the cDNA strand. The PCR amplification conditions were as follows: pre-denaturation at 95 °C for 10 min, denaturation at 95 °C for 10 s, annealing and extension at 60 °C for 30 s, and 40 cycles. Using β-Actin as an internal reference, the relative expression of the target gene was calculated using the 2− ΔΔCt method. The primers are shown in Table 1.

**Table 1:** The primers for qPCR analysis.

Target gene	Forward primer	Reverse primer
β-Actin	TGTCACCAACTGGGACGATA	GGGGTGTTGAAGGTCTCAAA
MMP7	GCTCTCAGAATGTGGAGTATG	CCCTTGCGAAGCCAATTA
AKT1	CTGTTCGAGCTCATCCTAATG	CTCTGTGTAGGGTCCTTCTT
CASP3	CGCCATGCTGAAACTGTA	CAGGGAGAAGGACTCAAATTC
MAPK3	GGACCTCATGGAGACGGACCTG	CGGAGGATCTGGTAGAGGAAGTAGC
VEGFA	CTACCAGCGCAGCTATTG	CAGGACGGCTTGAAGATATAC
HSP90AA1	CTACTGCACCAGAATGAAGG	GTTCCACAAAGGCTGAGTTA

### Histological analysis

The rat lung tissues were harvested and immediately fixed in a 4% paraformaldehyde (PFA) solution. After fixation, the tissues were dehydrated and embedded in paraffin, and 5-μm-thick sections were cut. The sections were then stained with Hematoxylin–Eosin (H&E) for general histological assessment and with Masson’s trichrome for collagen detection.

### Immunohistochemical detection

The antibodies used for immunohistochemical detection were MMP7 (GR231168-1, Abcam, Shanghai, China), HSP90AA1 (334,485, Abmart, Shanghai, China), Caspase3 (76i4559/6, Affinity Biosciences, OH, USA), AKT1 (20t9742, Affinity Biosciences, OH, USA), MAPK3 (B1601, Immunoway, Suzhou, China), and VEGFA (AC2111006A, Servicebio, Wuhan, China). The immunohistochemical procedure followed the standard protocol for tissue staining, including deparaffinization, antigen retrieval, blocking, primary antibody incubation, secondary antibody incubation, DAB development, counterstaining, dehydration, and mounting. The gray value analysis was carried out by randomly selecting 7 fields of each slice (magnification, 200x), and the mean optical density (MOD) of each field was measured using Image-Pro Plus 6.0 software. In order to automatically identify and select DAB-positive stained areas while excluding the influence of nuclei and other non-specific staining, we set an appropriate color threshold range based on the color characteristics of DAB staining. In the HSI color space, we set the Hue from 0 to 30, Saturation from 0 to 255, and Intensity from 0 to 220. Next, we used the color threshold function of the software to perform binary processing of the image according to the set color range. This process accurately identified DAB-positive stained areas while excluding cell nuclei and other non-specific stained regions. To further optimize the identification results, we applied morphological operations to the binary image, including opening (erosion followed by dilation) and closing (dilation followed by erosion), to remove noise and fill gaps. Finally, after completing the image processing, we calculated the MOD to assess the differences in protein expression levels between the groups, with higher MOD indicating higher expression levels of the target protein.

## Statistical analysis

All data were analyzed with GraphPad Prism v9.4.1 and were presented as means ± SD. Shapiro–Wilk tests were performed to determine the normality of the data distribution. For normally distributed data, one-way ANOVA was used to analyze the differences between groups. For data not conforming to a normal distribution, Kruskal–Wallis analysis was used. The significance levels were defined as follows: *, *P* < 0.05; **, *P* < 0.01; and ****, *P* < 0.001.

## Results

### The DEGs of IPF

**Figure 2:**
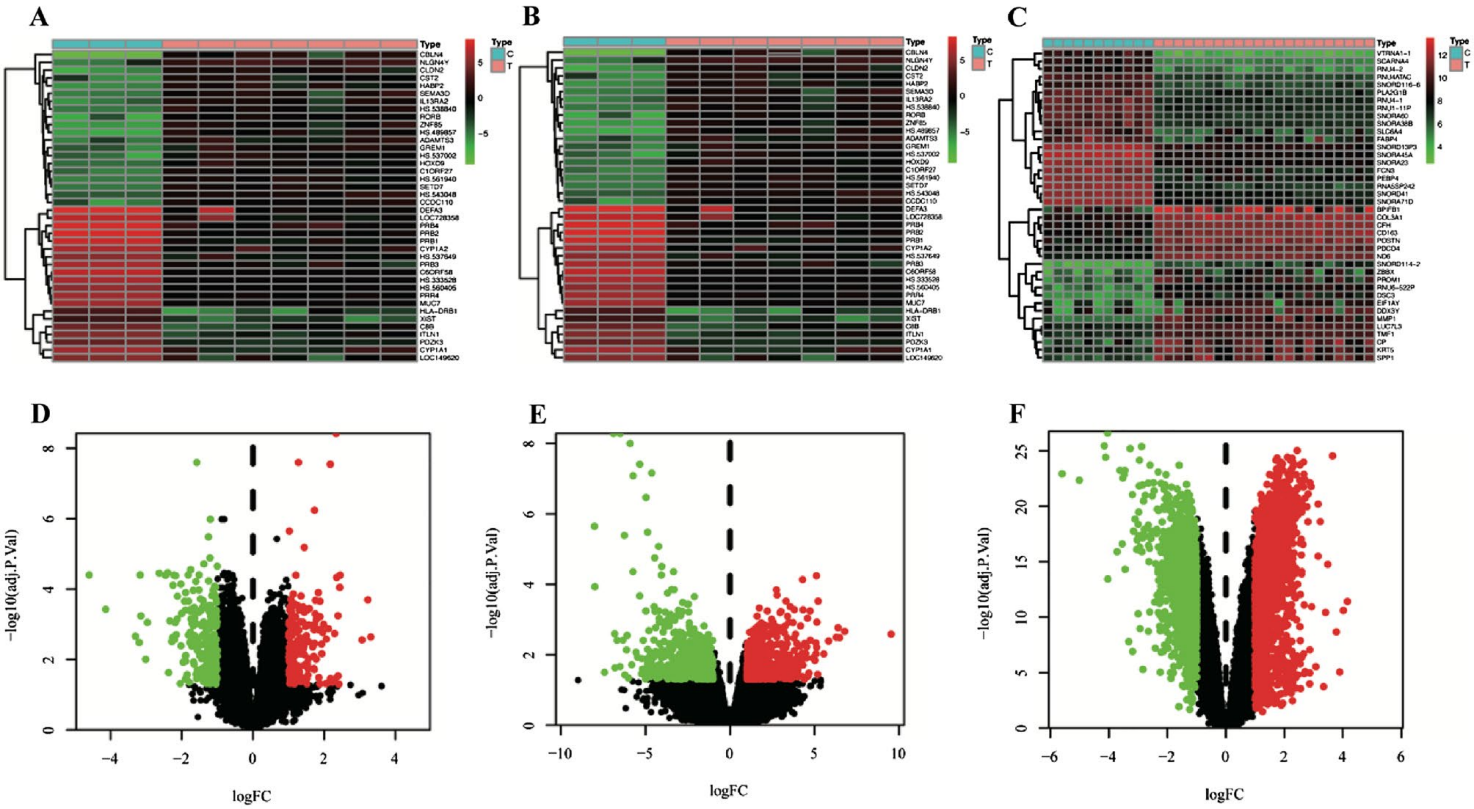
Heatmap of DEGs in (**A**) GSE24206, (**B**) GSE101286 and (**C**) GSE110147 (Created with TBtools v1.09876.3, https://github.com/CJ-Chen/TBtools). (**D**) Volcano plot of DEGs in GSE24206. (**E**) Volcano plot of DEGs in GSE101286. (**F**) Volcano plot of DEGs in GSE110147.

A total of 4640 DEGs were identified in lung tissues of IPF patients compared with normal lung tissues (Supplement Table S1). In the GSE24206 dataset, 227 genes were down-regulated, and 185 were up-regulated (Fig. 2A,D). In the GSE101286 dataset, 502 genes were down-regulated, and 571 were up-regulated (Fig. 2B,E). In the GSE110147 dataset, 1293 genes were down-regulated, and 2238 were up-regulated (Fig. 2C,F). The mean (SD) age of IPF patients in the GSE24206, GSE101286, and GSE110147 datasets was 67.35 (5.00), 66.75 (6.50), and 62.00 (6.00), respectively. Considering that IPF is an age-related disease, all patients are age-matched in these datasets.

### Bioactive ingredients and targets screening

Among the 87 and 119 chemical ingredients of AM and RPR obtained from TCMSP, 15 ingredients of AM and 13 of RPR with OB ≥ 30% and DL ≥ 0.18 were screened out. Despite not meeting the inclusion criteria, Astragaloside IV (OB = 17.7, DL = 0.15), Astragaloside III (OB = 31.8, DL = 0.10), Astragaloside II (OB = 46.1, DL = 0.13), and Astragaloside I (OB = 46.8, DL = 0.11) were included in this study for their bioactivities reported in previous studies. Finally, 32 bioactive ingredients were included, as shown in Table 2. A total of 728 genes were identified as potential targets of AM and RPR from SwissTargetPrediction after deleting duplicates (Supplement Table 2). By matching DEGs of IPF and targets of AM and RPR, 171 genes were selected as potential targets in the therapeutic effect of AM and RPR in IPF.

**Table 2:** Bioactive ingredients of AM and RPR.

Mol. ID	Molecular name	OB	DL
Astragalus membranaceus			
MOL000442	3,4-(4-Methoxy-6-hydroxy-1,2-phenyleneoxy)-5-hydroxy-7-methoxy-2H-1-benzopyran	39.1	0.48
MOL000439	Isomucronulatol-7,2′-di-O-glucosiole	49.3	0.62
MOL000438	(R)-Isomucronulatol	67.7	0.26
MOL000433	Folic Acid	69.0	0.71
MOL000422	Kaempferol	41.9	0.24
MOL000417	Calycosin	47.8	0.24
MOL000398	Isoflavanone	110.0	0.3
MOL000392	Formononetin	69.7	0.21
MOL000387	Bifendate	31.1	0.67
MOL000380	Astrapterocarpan	64.3	0.42
MOL000379	Methylnissolin-3-O-glucoside	36.7	0.92
MOL000378	7-O-methylisomucronulatol	74.7	0.3
MOL000371	3,9-di-O-methylnissolin	53.7	0.48
MOL000354	Isorhamnetin	49.6	0.31
MOL000098	Quercetin	46.4	0.28
MOL000409	Astragaloside IV	17.7	0.15
MOL000407	Astragaloside III	31.8	0.1
MOL000403	Astragaloside II	46.1	0.13
MOL000401	Astragaloside I	46.8	0.11
Radix paeoniae rubra			
MOL001921	Lactiflorin	49.1	0.8
MOL001924	Paeoniflorin	53.9	0.8
MOL007004	Albiflorin	30.3	0.8
MOL000449	Stigmasterol	43.8	0.8
MOL004355	Spinasterol	43.0	0.8
MOL002776	Baicalin	40.1	0.8
MOL000358	β-sitosterol	36.9	0.8
MOL007003	Benzoylpaeoniflorin	31.1	0.5
MOL001002	Ellagic acid	43.1	0.4
MOL007016	Paeoniflorigenone	65.3	0.4
MOL000492	Cianidanol	54.8	0.2
MOL002714	Baicalein	33.5	0.2
MOL002883	Ethyl oleate	32.4	0.2

### Protein–protein-interaction network construction

**Figure 3:**
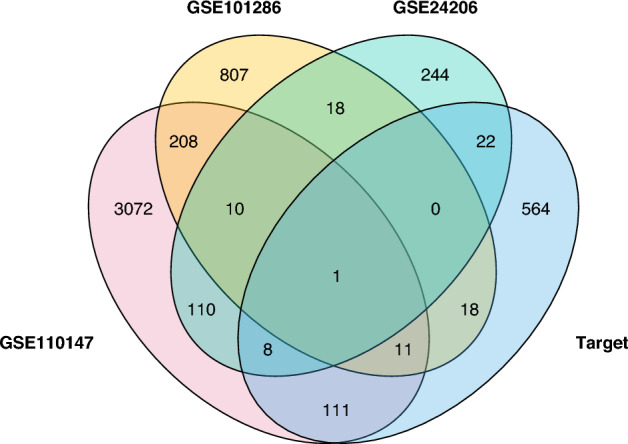
Common targets between DEGs of IPF and target of drugs.

**Figure 4:**
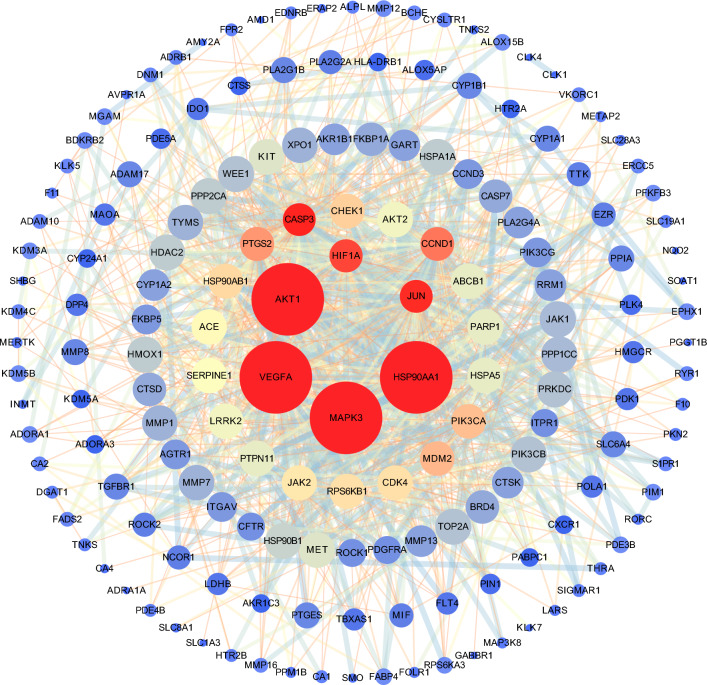
PPI network. The node size and color are proportional to its degree value. The larger and brighter the node, the more important the target in the network.

A Venn diagram was used to obtain intersections between targets of AM, RPR, and DEGs of IPF (Fig. 3). 171 common targets were identified, and based on these targets, a PPI network was constructed. The network consisted of 166 nodes and 1030 edges after setting confidence level > 0.4 (Fig. 4). In this network, each node represents a target, and each edge represents the interaction between two targets. The larger the size of the node, the brighter the color, and the closer to the center of the circle, the higher the degree value of the node, which means that there are more nodes connected to it; the thicker the edge and the brighter the color, the higher the betweenness of the edge. *AKT1*, *MAPK3*, *HSP90AA1*, *VEGFA*, *CASP3*, *JUN*, *HIF1A*, *CCND1*, *PTGS2*, and *MDM2* were predicted as the key targets according to the descending order of degree values (Supplement Table S3).

### GO/KEGG enrichment analysis and component-target-pathway network

**Figure 5:**
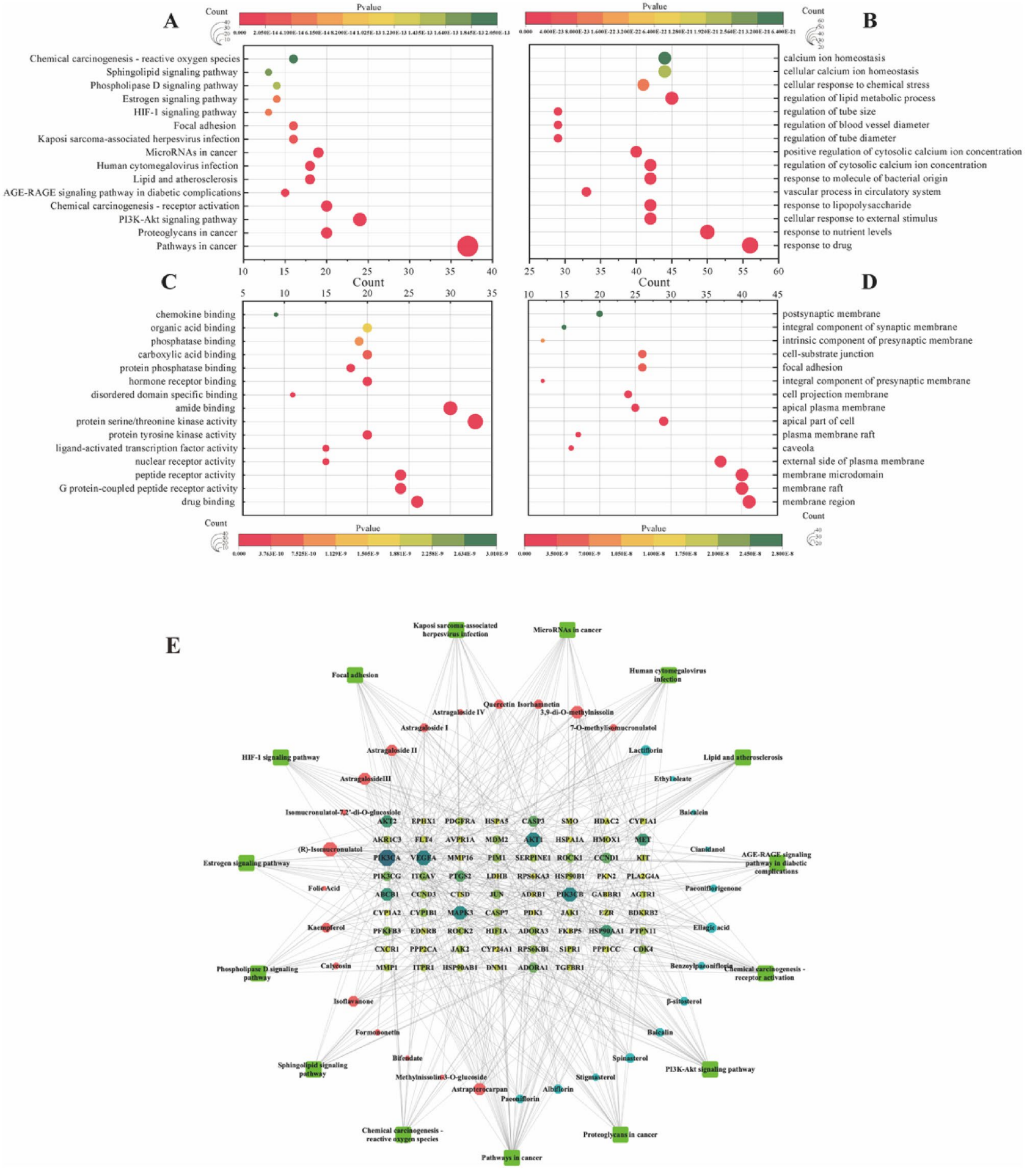
(**A**) KEGG analysis.^33–35^ (**B**) GO-BP analysis. (**C**) GO-MF analysis. (**D**) GO-CC analysis. (**E**) CTP network. The outer circle of the network represents the top 15 pathways; the inner circle represents components of AM and RPR that are related to genes enriched in these pathways; the innermost grid layout represents each target gene. Greater size of nodes represents higher degree value.

GO and KEGG enrichment analysis were performed on the 171 common targets using the Metascape platform (Supplement Table S4). A total of 173 KEGG pathways were significantly enriched, involving inflammation, apoptosis, and cell survival, such as the PI3K-AKT signaling pathway, HIF-1 signaling pathway, apoptosis, and MicroRNAs in cancer (Fig. 5A). Additionally, GOBP analysis revealed that AM and RPR could affect the response to drugs, response to nutrient levels, cellular response to external stimuli, cellular response to lipid metabolic processes, and so on (Fig. 5B). According to the ascending order of *P*-values, the top 15 GOMF and GOCC items were also shown in Fig. 5C,D. The CTP network contained 116 nodes and 502 edges (Fig. 5E). In this network, the stress of Astragaloside III, (R)-Isomucronulatol, Astragaloside I, Paeoniflorin, and β-sitosterol was 19,660, 17,304, 5622, 5112, and 3910, respectively. These compounds were considered as key ingredients in the anti-IPF effect of AM and RPR. These results suggest that the AM and RPR treatment may have a therapeutic effect on IPF through multiple targets and pathways. The key ingredients identified, such as Astragaloside III, (R)-Isomucronulatol, Astragaloside I, Paeoniflorin, and β-sitosterol, could be crucial components in mediating the anti-IPF effects of the AM and RPR. The enrichment analysis also highlights the potential roles of these compounds in modulating various biological processes, molecular functions, and cellular components, further supporting their potential therapeutic value in IPF treatment.

### Molecular docking

**Figure 6:**
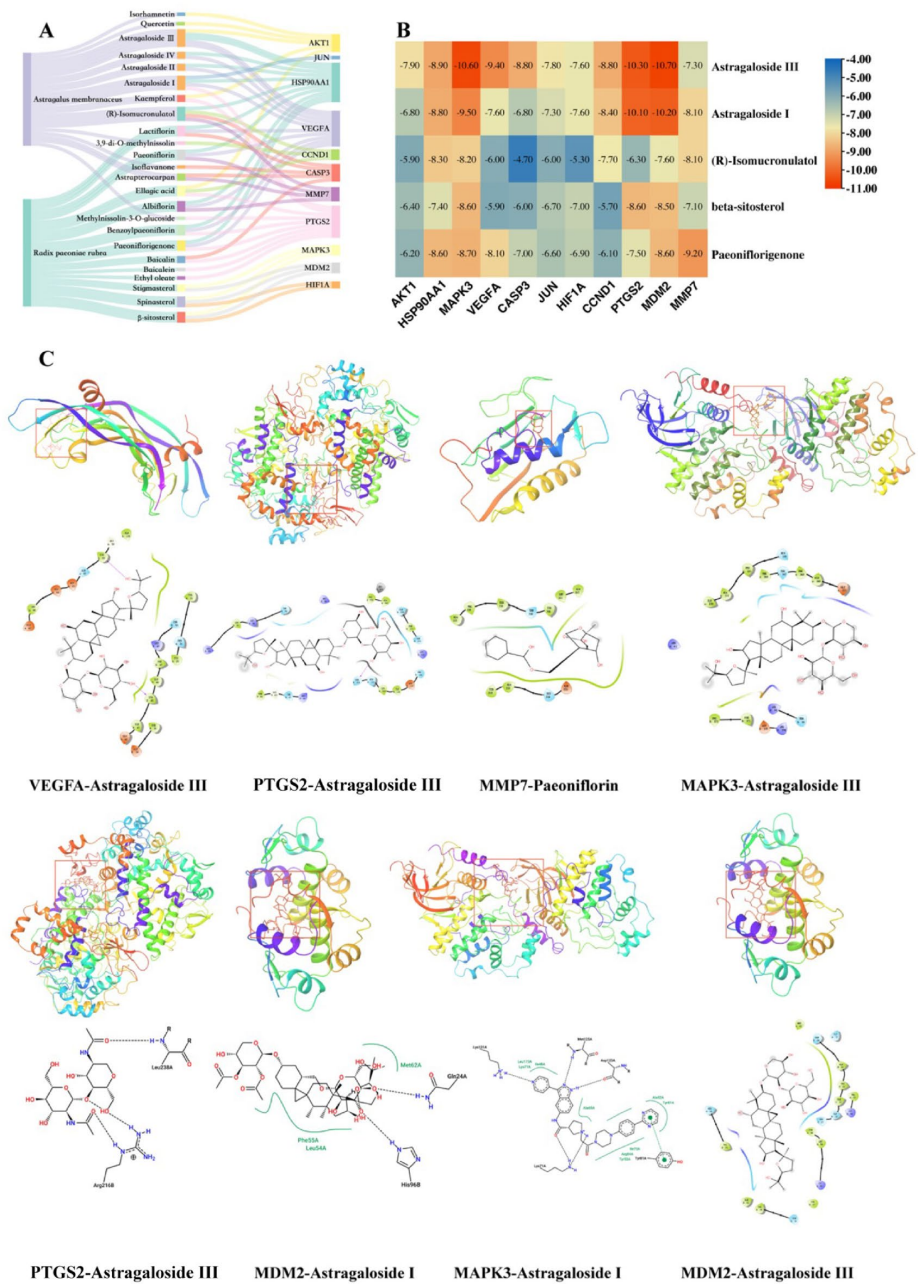
(**A**) Sankey diagram of showing the correspondence between 2 Chinese medicines, 24 components, and 11 targets. AM: astragalus membranaceus; RPR: Radix paeoniae rubra. (**B**) Heatmap of molecular docking score (Created with TBtools v1.09876.3, https://github.com/CJ-Chen/TBtools). (**C**) The molecular docking complexes. The binding site of the ligand and the protein residue has been identified in 2D form.

In the Venn diagram, MMP7 was the only overlapping target of all parts. As a result, 10 key targets and MMP7 were selected as receptors in the process of molecular docking. The one-to-one correspondence between the 11 targets and active components was visualized with a Sankey diagram, which was the basis for further molecular docking between the five most important components and 11 targets (Fig. 6A).

The molecular docking scores are shown in the heat map in Fig. 6B. The docking scores ranged from − 4.7 to − 10.7 kcal/mol, indicating a good binding affinity between the main active compounds and key targets. The docking processes of each component and the protein encoded by the key genes with a docking score less than − 9 kcal/mol were visualized (Fig. 6C).

These findings suggest that the active components of AM and RPR have a strong binding affinity to the key targets associated with IPF. The molecular docking analysis further supports the potential of these compounds to interact with key targets and modulate their activity, which may contribute to the therapeutic effects of AM and RPR in treating IPF. This provides valuable insights into the molecular mechanisms by which AM and RPR may exert their beneficial effects in IPF treatment and highlights the importance of further experimental and clinical research to validate these findings.

### The potential AM and RPR alleviated pulmonary fibrosis in BLM rats

**Figure 7:**
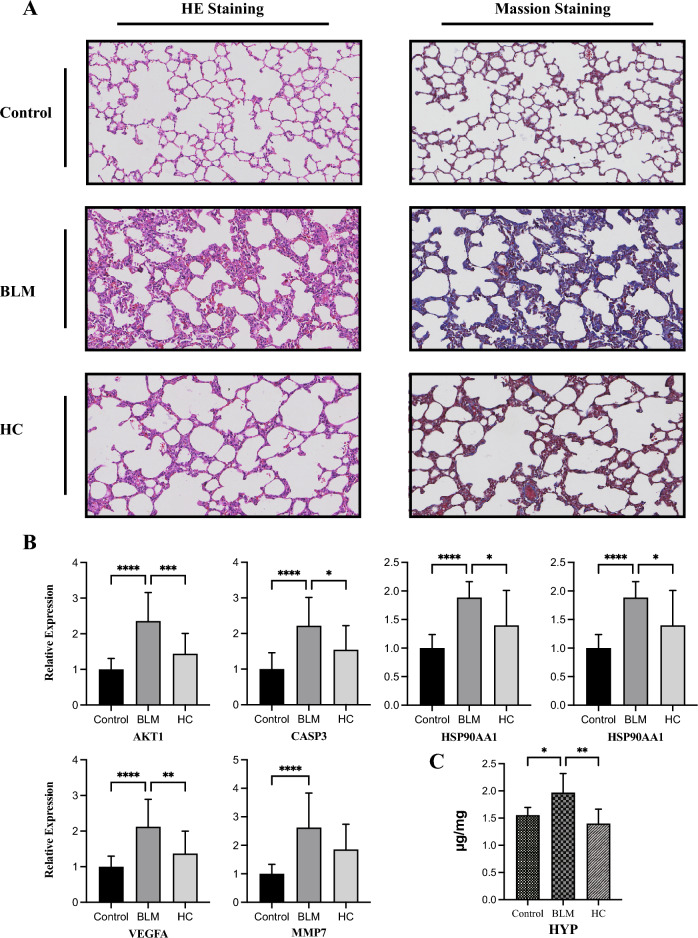
(**A**) Representative images of three groups stained with H&E and Masson's trichrome (magnification, 200×) (**B**) The mRNA expression of 6 targets screened out by network pharmacology (**C**) HYP levels in lung tissues.

The in vivo experimental validation provided further evidence supporting the therapeutic effects of AM and RPR on IPF. The histological analysis revealed that AM and RPR treatment alleviated BLM-induced pulmonary fibrosis in the rat model, as evidenced by the improvement in alveolar structure and reduced fibrosis compared to the BLM group (Fig. 7A).

The qPCR analysis of the six key targets (*AKT1*, *HSP90AA1*, *CASP3*, *MAPK3*, *VEGFA*, and *MMP7*) previously identified in the network pharmacology study provided insights into the potential molecular mechanisms underlying the therapeutic effects of AM and RPR on IPF. The expression levels of *AKT1*, *HSP90AA1*, *CASP3*, *MAPK3*, *VEGFA*, and *MMP7* were significantly increased in the BLM group compared to the control group (Fig. 7B). However, treatment with AM and RPR attenuated the increased expression of these targets, with *MMP7* showing a downward trend upon treatment.

**Figure 8:**
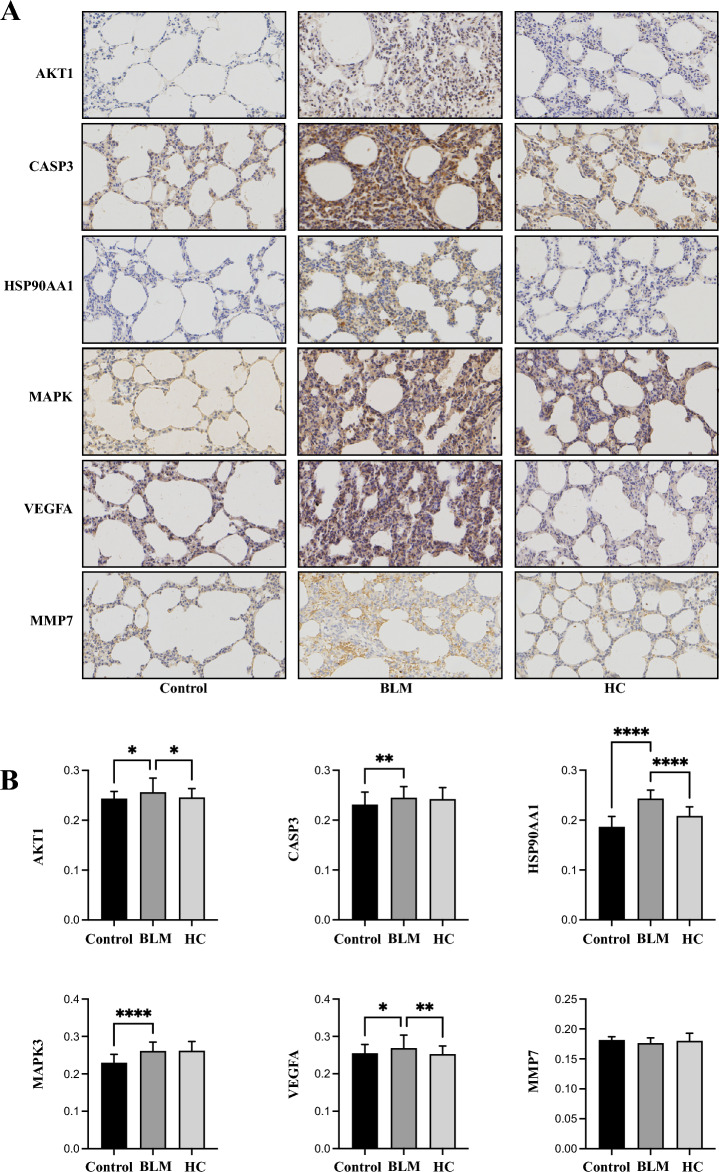
(**A**) Immunohistochemical staining of lung tissue sections (**B**) Semi-quantitative analysis of six key targets.

HYP is predominantly found in collagen, a major component of the extracellular matrix deposited during fibrosis. In bleomycin-induced fibrosis, there was an increasing trend in HYP levels compared to the control group, though it was not statistically significant. However, in the HC group, the HYP levels significantly decreased compared to the BLM group (Fig. 7C). To confirm the protein expression levels of the six key targets in lung tissue, we performed immunohistochemical analysis (Fig. 8A). The MOD of each group was compared (Fig. 8B). AKT1-positive products were located in the nucleus, and the expression of AKT1 in the BLM group was significantly higher than that in the control group. Conversely, the expression of AKT1 protein in the HC group was significantly lower than the BLM group. CASP3 protein was primarily expressed in the cytoplasm, and its expression in the BLM group was significantly upregulated compared to the control group, with a statistically significant difference. There was no significant difference in CASP3 protein levels between the HC and BLM groups. HSP90AA1-positive products were located in the cytoplasm and plasma membrane, and the expression level of HSP90AA1 significantly increased in the BLM group but decreased in the HC group. MAPK3-positive products were localized in the nucleus, and following bleomycin treatment, the expression levels of MAPK were significantly elevated, with no notable differences observed between the HC group and the BLM group. VEGFA proteins were observed in the plasma membrane, cytoplasm, and nucleus. The level of VEGFA was elevated in the BLM group compared to the control group, and it was significantly reduced in the HC group. Furthermore, MMP7 protein was mainly expressed in the plasma membrane and extracellular matrix, and its expression was similar across the three groups, with no significant differences observed.

## Discussion

IPF, the most severe subtype of interstitial lung disease, is characterized by chronic inflammation, extracellular matrix (ECM) deposition, epithelial-mesenchymal transition (EMT) dysfunction, and abnormal lung tissue structure remodeling^36^. Currently, there is no widely accepted effective therapeutic approach for IPF, necessitating the urgent development of novel, effective, and safe treatments. Natural products have become increasingly important resources for pharmaceutical research and development due to their unique biological functions. AM and RPR are two primary ingredients in Buyang Huanwu Decoction (BYHWD), a renowned Chinese formula for supplementing Qi and activating blood circulation. A recent study demonstrated the protective effect of BYHWD on a pulmonary fibrosis model in vitro, with its mechanism related to the inhibition of pulmonary inflammation, collagen deposition, and EMT by suppressing the TGF-β1 signaling pathway^37^. An AM extract injection has been reported to ameliorate pathological lung fibrotic damage caused by bleomycin in rats and improve lung function by reducing the expression levels of TGF-β1 and collagens I and III, while increasing those of *MMP-3*, *MMP-9*, *TIMP-1*, *CXCL12*, and *CD90*^38^.

Consequently, we employed network pharmacology analysis to investigate the common transcription factor regulatory network in IPF, identifying AM and RPR as potential IPF treatments. The therapeutic effects and mechanisms of AM and RPR were verified in vivo, while network pharmacology analysis revealed their molecular functions and pharmacological targets for IPF treatment. The diverse and heterogeneous etiology and pathogenesis of IPF make it suitable for identifying pathways and targets through comparison of samples from IPF patients and control tissues based on microarray screening. Initially, 171 overlapping targets were identified among targets of AM, RPR, and differentially expressed genes (DEGs) in GSE110147, GSE101286, and GSE24206. Using these 171 targets, we revealed a regulatory network and key targets in IPF regulation. Subsequently, GO and KEGG enrichment analyses were performed, indicating that multiple biological processes and pathways contribute to the anti-IPF effects of AM and RPR. These pathways are associated with inflammation, autophagy, apoptosis, and cell survival, including the PI3K/AKT pathway, microRNAs in cancer, and apoptosis, among others. The PI3K/AKT pathway is implicated in numerous IPF-related pathological changes, such as alveolar epithelial cell damage, extracellular matrix overproduction, epithelial-mesenchymal transition, and apoptosis^39–42^. Targeting the PI3K/AKT pathway has already demonstrated benefits in IPF treatment^43^. However, extensive crosstalk and interactions between PI3K/AKT and other pathways, including TGF, VEGF, WNT, and Notch signaling pathways, suggest a complex IPF pathogenesis network^44^. Blocking a single node in this network may prove insufficient or ineffective.

The use of natural products like AM and RPR, which can act on multiple targets and pathways, may provide a more promising approach for the treatment of IPF. Astragaloside III, (R)-Isomucronulatol, Astragaloside I, Paeoniflorin, and β-sitosterol were selected as the main active components according to the CTP network and Sankey diagram. Numerous metabolites of AM and RPR have already been proven to be promising agents for alleviating fibrosis progression in multiple organs. Astragaloside IV has been shown to significantly inhibit BLM-induced EMT in pulmonary fibrosis by targeting the PI3K/AKT pathway^45^. Quercetin has been demonstrated to counteract ROS damage and inflammation in the process of IPF^46^. Paeoniflorin has been found to inhibit the early stages of TGF-β-mediated EMT in alveolar epithelial cells by decreasing the expression of the transcription factor Snail through the upregulation of Smad7^47^. Molecular docking validation was conducted, and the main active components were predicted to exhibit strong binding affinity with key targets.

Based on these findings, we further investigated the therapeutic effects of AM and RPR on IPF. In the experimental validation phase, we discovered that AM and RPR could alleviate pulmonary alveolar damage and, to some extent, reduce fibrosis. We then conducted quantitative measurements of hydroxyproline (HYP) and found that bleomycin tended to increase HYP content in lung tissue, while AM and RPR intervention led to a significant decrease in HYP, demonstrating their anti-fibrotic effects. qPCR revealed increased expression of *AKT1*, *HSP90AA1*, *CASP3*, *MAPK3*, *VEGFA*, and *MMP7* after bleomycin treatment, consistent with the results of previous studies.

Mitophagy and apoptosis exhibit cell type-specific features in the progression of pulmonary fibrosis. Apoptosis and mitophagy-induced type II alveolar epithelial cells, along with apoptosis-resistant and mitophagy-impaired macrophages, synergistically promote the development of pulmonary fibrosis. Several transcription factors contribute to these processes. *AKT1* activation plays a crucial role in regulating pulmonary fibrosis, as it is strongly associated with the survival and differentiation of myofibroblasts. The function of *AKT1* in the progression of pulmonary fibrosis is closely related to *TGF-β*. In alveolar macrophages, *TGF-β* induces *AKT1* activation, which promotes mitochondrial reactive oxygen species (ROS) and mitophagy. This *AKT1*-mediated mitophagy leads to apoptosis resistance and prolonged survival of macrophages, which are necessary for pulmonary fibrosis progression^48^. However, apoptosis has the opposite effect in alveolar epithelial cells. Apoptosis of alveolar cells contributes to early fibrosis and lung injury. Increased levels of pro-apoptotic factors, such as Caspase-3, Bax, and PARP, have been observed in lipopolysaccharide-induced pulmonary fibrosis models, and induction of alveolar cell apoptosis can exacerbate pulmonary fibrosis^49^.

Additionally, the activation of non-Smad signaling pathways, including *MAPK* and *AKT* by *TGF-β1*, promotes aspects of EMT^50^. The activation of the PI3K/AKT pathway has been identified to inhibit the function of *FOXO3*, and the repressed expression of *FOXO3* in normal fibroblasts contributes to the IPF fibroblast phenotype^51^. *HSP90*, a member of stress-inducible proteins, consists of two isoforms, *HSP90A* and *HSP90B*, and participates in the regulation of *TGF-β1* by inhibiting the activity of CHIP (carboxyl terminus of Hsc70-interacting protein) to ubiquitinate and degrade Smad3^52^. *HSP90* inhibition has been proven to abrogate TGF-β-induced fibroblast activation and ECM production^53^. Several MAPKs, including *ERK*, *JNK*, and *p38*, have been shown to activate activation protein 1 (*AP-1*), which is involved in the phenotypic transformation of human lung fibroblasts into myofibroblasts induced by *TGF-β*. *VEGFA* is a permeability and angiogenic factor. A recent study demonstrated that the level of *VEGFA* correlated with the *TGF-β* level and could be raised by bleomycin, which is consistent with our results^54^.

Matrix metalloproteinases (*MMPs*) are metalloendopeptidases that can degrade components of the ECM and non-matrix proteins. Most *MMPs* have been shown to promote the development of IPF and are upregulated in IPF blood and/or lung samples. Although *MMP7* is not the key target predicted by the PPI network, it is a DEG in all three series and a potential target of AM and RPR. The role of *MMP7* in IPF includes promoting EMT, increasing lung levels or activity of profibrotic mediators, and reducing lung levels of antifibrotic mediators^55^.

We subsequently performed immunohistochemical assays to measure the protein levels of the aforementioned targets in lung tissues. The results generally aligned with the PCR findings: BLM treatment induced the expression of *AKT1*, *CASP3*, *HSP90AA1*, *MAPK*, and *VEGFA*, while drug treatment reduced their expression. However, we did not observe significant differences in *MMP7* levels among the three groups, despite previous research suggesting that bleomycin injury triggers lung MMP7 overexpression^52^. Such inconsistency may be related to the limitations of semi-quantitative analysis. The downward trend of these factors after treatment implies a multi-target and multi-pathway characteristic of AM and RPR in anti-IPF treatment. We speculate that AM and RPR suppressed TGF-β1/PI3K/Akt, MAPK, and VEGF signaling pathways by targeting these pharmacological targets, subsequently ameliorating ECM deposition and inflammation in IPF. It is noteworthy that the research findings suggest AM and RPR may reduce inflammation in the BLM-challenged rats; however, inflammation is not the pathogenic mechanism underlying IPF. IPF is not an inflammatory disease, and although minor regions with inflammatory cells may be present, it does not undergo a transition from an inflammatory to a fibrotic phase. Moreover, IPF patients do not experience significant improvement following anti-inflammatory or corticosteroid treatments. Consequently, when discussing the potential of plant-derived compounds in targeting pharmacological agents to alleviate ECM deposition and inflammation in IPF, it is crucial to recognize that reducing inflammation in IPF would not enhance patients' quality of life; instead, ECM accumulation represents the genuine issue in IPF^7^.

Some limitations exist in this study. Certain active components of Chinese medicine may change during the decoction process, while other compounds with therapeutic effects that may be generated during the process are not included in this study. Additionally, we investigated several nodes in the complex network of multiple targets and pathways; further studies could link the upstream and downstream transcription factors of key targets to more clearly reveal the regulatory role of AM and RPR in the signaling pathways involved in IPF. It is anticipated that future researchers will overcome challenges and translate the extensive understanding of IPF pathogenetic mechanisms into effective therapeutic approaches.

## Conclusion

In this study, we investigated the potential pharmacological targets and therapeutic mechanisms of AM and RPR on IPF using network pharmacology and verified our findings through in vivo experiments. In conclusion, this study identified AM and RPR as potential therapeutic agents for IPF by regulating *AKT1*, *HSP90AA1*, and *VEGFA*.

## Supplementary Information

Supplementary Tables.

## Data Availability

The datasets generated and/or analyzed during the current study are available in the Gene Expression Omnibus (GEO, https://www.ncbi.nlm.nih.gov/geo/) (Accession Number: GSE24206, GSE101286 and GSE 110,147), and the Worldwide Protein Data Bank (wwPDB, https://www.rcsb.org/) (Accession Number: 1UNQ, 4JJE, 2W96, 5L9B, 4BQG, 5VPB, 6GES, 4IPF, 2Y6D, 1PPX and 1mjv). The original data presented in the study are included in the article/Supplementary Material. Further inquiries can be directed to the corresponding authors.

## Abbreviations

IPF: Idiopathic pulmonary fibrosis
TCM: Traditional Chinese medicine
AM: Astragalus membranaceus
RPR: Radix paeoniae rubra
BLM: Bleomycin
GEO: Gene expression omnibus
TCMSP: Traditional Chinese medicine database and analysis platform
OB: Oral bioavailability
DL: Drug-likeness
PPI: Protein–protein interaction
DEGs: Differentially expressed genes
GO: The gene ontology
KEGG: Kyoto encyclopedia of genes and genomes
MF: Molecular functions
CC: Cellular components
BP: Biological processes
CTP: Component-target-pathway
*AKT1*: RAC-alpha serine/threonine-protein kinase
*MAPK3*: MAP kinase-activated protein kinase 3
*HSP90AA1*: Heat shock protein HSP 90-alpha
*VEGFA*: Vascular endothelial growth factor A
*CASP3*: Casparian strip membrane protein 3
*JUN*: Transcription factor Jun
*HIF1A*: Hypoxia-inducible factor 1-alpha
*CCND1*: G1/S-specific cyclin-D1
*PTGS2*: Prostaglandin G/H synthase 2
*MDM2*: E3 ubiquitin-protein ligase Mdm2
